# Children’s emerging concepts of resilience: insights from using body mapping in an East London cohort sample of 7-10-year-old children

**DOI:** 10.3389/fpsyg.2024.1408771

**Published:** 2025-01-06

**Authors:** Aisling Murray, Dominie Mahala Smith Scott, Milena Nikolajeva, Daniele Porricelli, Francois van Loggerenberg, Dennis Ougrin, Jennifer Y. F. Lau

**Affiliations:** ^1^Youth Resilience Unit, Academic Unit, Centre for Psychiatry and Mental Health, Wolfson Institute of Population Health, Queen Mary University of London, London, United Kingdom; ^2^University Hospitals Sussex NHS Foundation Trust, Princess Royal Hospital, Haywards Heath, Sussex, United Kingdom; ^3^Research and Development (R&D), North East London NHS Foundation Trust, Goodmayes Hospital Site, London, United Kingdom; ^4^Unitat de Recerca, Docència i Innovació, Parc Sanitari Sant Joan de Déu, Sant Boi de Llobregat, Spain

**Keywords:** resilience, qualitative methods, middle childhood, socioecological model, arts-based methods

## Abstract

**Background:**

Understanding resilience factors in children is essential for developing early mental health interventions. Middle childhood is an understudied developmental stage, with many quantitative measures lacking validation for this age group and not capturing diverse experiences. This study aimed to use body mapping, an arts-based method, as a novel approach to understand 7-10-year-old children’s concepts of resilience (including definitions and factors that contribute to resilience) in East London. An advisory group of six children commented on the findings.

**Methods:**

Body mapping was included in the Development of Emotional Resilience (DEER) Study. Participants drew a resilience symbol, wrote recent worries and colored on an A4-sized body map to signal where they embody stress. Demographic data were collected via self- and parent-report surveys and school records. Manifest content analysis identified four thematic categories related to worries, somatic stress and resilience.

**Results:**

196 children (48.47% boys, 46.43% girls; 35.20% White, 30.10% Asian, 11.22% Black) across school years 3–5 completed body mapping. Concepts of resilience included perseverance and metaphorical representations of personal strength. We also identified socioecological factors that contributed to resilience, mainly at the individual and interpersonal levels. Boys more often depicted Sports whilst more girls depicted Engagement in the arts and Social networks. 11 worry categories emerged, including education, relationships and physical health. Of the body categories colored (*n* = 51), the most common were the head, hands and abdomen/stomach.

**Conclusion:**

Children expressed dominant and abstract symbols of resilience and identified factors that contributed to resilience. Hobbies and strong relationships may be particularly important in middle childhood, corroborated by the advisory group’s experiences. Body mapping revealed diverse worries (e.g., education, change and uncertainty and global and societal concerns) and somatic experiences of stress (e.g., the head, chest and torso). Through prioritising children’s perspectives, body mapping holds promise in clinical and educational settings.

## 1 Introduction

Exacerbated by the COVID-19 pandemic (Racine et al., 2021; Kauhanen et al., 2023), the rates of mental health disorders in children and young people (CYP) are at record highs (Houtrow et al., 2014). Good mental health and psychosocial wellbeing, i.e., emotional, psychological, social and collective wellbeing (Eiroa-Orosa, 2020; Martikainen et al., 2002), are important for lifelong health and quality of life (WHO, 2014). However, alongside other factors - e.g., biological and psychological - environmental factors impact the risk of mental illness and access to services and subsequent mental health outcomes (WHO, 2014; Alegría et al., 2018). Many children are therefore deemed ‘at-risk’ of poor mental health outcomes due to environmental vulnerabilities, ranging from acute stressors like abuse and war to chronic stressors like family conflict and economic inequalities (Rak and Patterson, 1996; Poole et al., 2017; Wright et al., 2019). However, contrary to this “monochromatic view” (Ungar, 2005, p. xvi) of children at-risk, many display resilience due to protective factors and internal and external resources (Carbonell et al., 2002; Marley and Mauki, 2019; Yule et al., 2019). Understanding how children cope with stressors is essential for early intervention strategies to mitigate the costly consequences of poor mental health.

**Figure 1:**
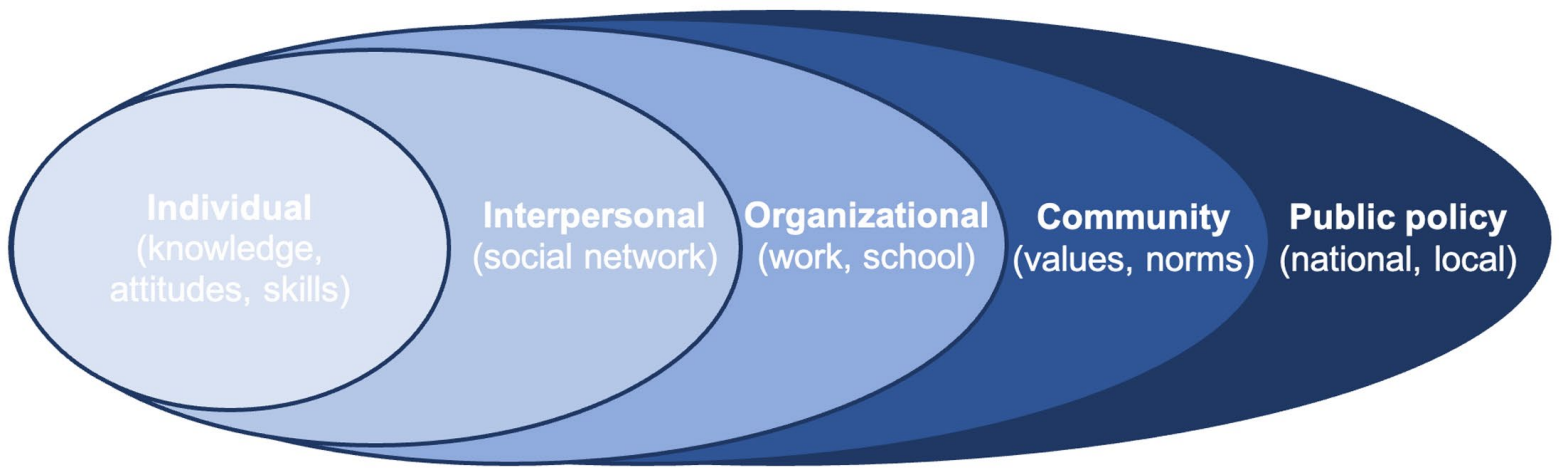
A diagram to show the five systems levels of the socioecological framework.

Resilience is broadly defined as the ability to overcome adversity and experience positive outcomes in spite of this adversity (Vella and Pai, 2019). Recent definitions have moved beyond trait-based concepts of stable invulnerability to stressors (Anthony and Cohler, 1987) to recognising resilience as an interactive relationship between individuals and communities and their environments (Ungar, 2008, 2011; Herrman et al., 2011; Ungar and Liebenberg, 2011; Theron, 2016; Vella and Pai, 2019). According to this theoretical framework, dynamic constructs such as resilience develop and are shaped not only by individual-level factors but also interactions with nested external factors, including family and peer groups in schools and the community (Bronfenbrenner, 1977, 1989). The socioecological framework therefore refers to specific social and environmental factors and their interactions across systems that protect CYP from the negative impact of adverse environmental conditions (Henderson et al. 2026; Theron, 2016). Situating resilience within a socioecological framework demonstrates the diverse pathways for interventions that support healthy development and promote resilience in CYP (Vaughn and DeJonckheere, 2021). Figure 1 visualizes these nested systems in which individuals are embedded.

As a lifelong process, it is important to consider resilience across these nested socioecological systems in the context of developmental stages (Ungar, 2012; Gartland et al., 2021). Middle childhood (5–12 years) is a critical, but understudied, stage of development compared with adolescence and adulthood (Windle et al., 2011; Gartland et al., 2021). Some key developmental tasks are pronounced in middle childhood, such as the development of emotional bonds with caregivers and the development of a sense of gender identity (Egan and Perry, 2001). In addition, this is a period of significant emotional development, including a rapid increase in self-regulation (Raffaelli et al., 2005) and distinct onset of psychological conditions, such as anxiety-related disorders (Costello et al., 2003; Kessler et al., 2005). Due to developmental differences in maturation, the factors contributing to resilience in middle childhood likely differ from those in later life (Gartland et al., 2019), highlighting the importance of research focused on this age group.

However, many existing approaches to measure resilience have not been tailored to children. For example, several measures of resilience were originally developed using data from adults and therefore lack content validity (Windle et al., 2011). Additionally, many quantitative measures were developed with White, middle-class Western adults and adolescents (Levesque, 2011), and so the experiences of children from diverse backgrounds are overlooked and not captured. Our current understanding of resilience also largely draws from parent and teacher reports (Shean, 2015), which similarly biases findings toward adult perceptions of children’s experiences. Even existing measures for children do not always capture middle childhood. For example, the Child and Youth Resilience Measure (Ungar and Liebenberg, 2011), which was co-produced with CYP, is yet to be validated for children below age 13. Whilst an inclusive measure of resilience in middle childhood is being developed (Gartland et al., 2021), there remains limited research on this stage of development.

Qualitative inquiry accounts for context and local definitions of positive outcomes (Ungar, 2003). Arts-based research (ABR) is a form of qualitative inquiry which uses symbolism, metaphor and artistic processes to explore subjective human experiences (Wimpenny and Savin-Baden, 2014). Given that children learn to use verbal language progressively, visual ABR methods use art to go “beyond logical verbal language to come to an understanding of voice” (Caldairou-Bessette et al., 2020, p. 3) which includes non-verbal expression (Teachman and Gibson, 2018). ABR also offers a palatable, non-stigmatising means of engaging with sensitive topics (Hickey-Moody et al., 2021), making it suitable for research with CYP. For example, in their systematic review of using ABR methods with young people with complex psychosocial needs, Nathan et al. (2023) found that these methods drew out young people’s strengths, resilience and optimism and enabled meaningful exploration of experiences.

Embodied inquiry, a research approach centred on embodied lived experiences, enables exploration of “deeper, emotional and authentic truths about lived experience” (Leigh and Brown, 2021, p. 27) than traditional research approaches. Body mapping is a visual ABR method rooted in embodied inquiry, which typically involves tracing the body to produce a life-sized outline then filled with words, colors and symbols to reflect embodied experiences (de Jäger et al., 2016). Since its inception in research to explore physical health (MacCormack and Draper, 1987; Solomon, 2002; MacGregor and Mills, 2011), it has grown as a research method to explore mental health and resilience, including that of children (Murray et al., 2023; Barnes et al., 2024). Body mapping offers an innovative approach to resilience research that overcomes several limitations of traditional research methods. Quantitative methods, such as standardized questionnaires, may not fully capture the dynamic and changeable nature of mental health, whilst verbal qualitative methods such as interviews may not suit unconventional, non-linear mental health narratives (Llewellyn-Beardsley et al., 2020). In contrast, body mapping provides a visual means for participants to communicate their experiences, capturing the dynamic and multifaceted nature of resilience. Finally, traditional qualitative methods may be subject to researcher bias, whilst body mapping is a participatory method that relies on participant involvement and ownership of the research process, limiting the risk of researcher bias (Boydell et al., 2018).

This exploratory study aimed to use body mapping to address two primary research questions on concepts of resilience for 7-10-year-old children in East London:

1. How do children represent and conceptualize resilience?
2. Do children’s concepts of resilience vary by gender?

We hope that these data will inform how children perceive resilience and the factors which promote it. We also addressed two secondary research questions to understand common stressors and how stress is embodied:

3. What are children’s reported sources of recent worries?
4. Where do children experience somatic symptoms of stress?

We posed these RQs in East London. With its young and diverse population, East London provides a unique setting to explore resilience in children who may be at-risk of poor mental health outcomes. Alongside increasing gentrification, higher housing and living costs continue to exacerbate health and social inequalities (Ucci et al., 2022). The region, which includes seven London boroughs, is characterized by high levels of child poverty, with Tower Hamlets (56%), Newham (50%) and Barking and Dagenham (48%) having the highest rates of all UK local authorities in 2019–20 (Joseph Rowntree Foundation, 2022). Whilst region-level data is limited, research has shown the interplay of cultural, familial and individual factors that influence mental health outcomes and resilience in CYP in East London (Stansfeld et al., 2004; Fagg et al., 2006).

## 2 Materials and methods

This study was embedded in the Development of Emotional Resilience (DEER) Study, an East London-based observational cohort study that uses an accelerated longitudinal design. The DEER Study was designed to understand the factors that influence children’s resilience trajectories by assessing children aged 7–11 annually with measures of relationships, emotional responses to situations, lifestyle variables and access to mental health support and community resources. Participants were recruited through schools and data collection took place in school classrooms with groups of 10–15 participants at a time. Parental consent and participant assent were sought, and participants could withdraw at any point or skip any measure (including body mapping). Our ethics approval, granted by the Queen Mary Ethics of Research Committee, included procedures for handling participant disclosures.

### 2.1 Demographics

Gender was self-reported during the assessment sessions. Ethnicity information was collected either from parents using an online survey (Qualtrics software, Prove, UT) or from schools using a physical or digital questionnaire. Ethnicity questions were based on Office for National Statistics (ONS) recommendations (ONS, n.d.). In addition, we report on participants’ Year Group, including Year 3 (7–8 years), Year 4 (8–9 years) and Year 5 (9–10 years).

### 2.2 Body mapping

This was included in the DEER Study assessment battery from October 2022 to March 2023 and data was collected from 196 participants across seven schools in five boroughs in East London. Body mapping complemented the other measures in the DEER study by providing a non-verbal form of expression and helping to ensure that participants continued to engage well with the assessment battery and did not experience survey fatigue (Britton et al., 2020). Body mapping took approximately 10–15 min for participants to complete (a blank version including instructions is shown in Supplementary material 1). The A4 sheet included (1) a body template for participants to color in red where they experience embodied stress, (2) a ‘recent worries’ bubble for participants to write any recent worries they have had and, (3) a space for participants to draw and describe a symbol to represent their interpretation of resilience. To ensure that children understood the task, a researcher from the data collection team (DP, MN, AM, DSS) explained the activity either to the whole group or to small groups of participants, depending on whether they had finished the other tasks at a similar time. The researcher went through the instructions as written on the A4 sheet to ensure consistency across groups. Of note, 34 participants completed an earlier version of body mapping using multiple colors to highlight embodied stress. However, this made interpretation of the locations of embodied stress more ambiguous, therefore we changed this to red only. These 34 participants’ data were not included in analysis relating to the location of embodied stress. Participants were provided with HB pencils and red colouring pencils for drawing and writing.

Children could provide as much information as they wished. As part of our safeguarding and distress protocol, if a participant became distressed during body mapping, they were told they could take a break or stop taking part if they wished, and the data collection team informed the school. If a researcher was worried about a participant’s safety after reviewing the content of their body map following data collection, they discussed this with the DEER Study principal investigators (JYFL, DO) and, if deemed necessary, informed the participant’s school in case further safeguarding action was required.

### 2.3 Analysis

We followed manifest content analysis, which involves describing the surface structure of participants’ responses and what is obvious from the data (Bengtsson, 2016). To develop the codes for each data category (worries, body parts and resilience symbols), AM and DSS looked over the data multiple times to gain familiarity. These researchers then independently inputted the data into NVivo 12, which involved transcribing the symbols and coloring data into textual descriptions. The researchers then met to compare the transcripts and develop accurate final versions of them. This ensured a standardized approach to preparing the body maps for analysis. Next, the researchers independently inductively coded a subset of the data, and met to ensure consensus in the coding strategies. The researchers then independently coded all the data and met to develop a final coding framework, with any differences discussed and resolved by agreement. The researchers then recoded the data based on this final coding framework. They met regularly to refine the coding framework and discuss emerging themes and categories (Bengtsson, 2016). During these discussions, the researchers referred back to the raw body map data if needed for clarification and deeper interpretation, particularly for the drawings. Final codes were grouped into themes and subthemes for the worries and resilience data, whilst the coloring data remained as singular codes such as ‘top of the head’. Final themes and descriptions for each were discussed and agreed upon between AM and DSS, with input from JYFL.

As an example of this process, for the resilience data, we initially created separate codes for support from parents and support from family members to distinguish specific depictions of parents from others, such as a ‘cousin’ and ‘my family’. However, we then combined this into a singular code of support from parents and other family members as all of this data related to receiving emotional support from family when feeling sad or worried. Along with other codes related to family and friendship, we then developed the theme social networks, which related to the category “Interpersonal level”. Table 1 displays examples of how codes, themes and categories were developed from the worries and resilience data.

**Table 1:** Selected examples of data analysis from transcripts to coding and developing themes and categories.

Data	Transcript	Code	Theme	Category
Worries	“I worry if I fail in exams and texts” “Parents’ evening is coming up and I’m worried whether my work is going to be as good as last year”	Academic achievement	Education	N/A
Resilience	Two stick people (one larger than the other) labeled “when I feel sad I like to talk to my family”	Support from parents and other family members	Interpersonal level (social networks)	Socioecological resilience factors
	A fish swimming with ‘you have to push through a problem so there is a fish who has to push through a current’ written.	Perseverance	Conceptual definitions	Concepts of resilience

Analysis was carried out on qualitative differences between boys and girls for resilience findings (RQ2). Given that few participants reported ‘other’ and ‘prefer not to say’ for their gender, we did not include participants of these categories of response in this comparison. Owing to relying on children’s drawing and writing, at times it was challenging to read or interpret participants’ responses. Therefore, any responses that were unclear, for example due to ambiguous intended meaning or difficulty reading handwriting, were coded as ‘unclear’. Worry or resilience responses that were missing were coded as ‘invalid’. Worry responses such as ‘no worries’ or ‘no’ were coded as ‘no worries’. Resilience responses which were negative, such as emotions like ‘angry’, ‘worried’, ‘sad’ or a worry being described, were coded as ‘negative descriptions’.

### 2.4 Reflexivity

Reflexivity is important to build an awareness of the relational and emotional aspects of research (Warin, 2011). The first author (AM) is a qualitative researcher with an existing interest in ABR methods, which may have impacted the design of the body mapping method. However, our multidisciplinary team, including researchers from psychology, psychiatry, medicine and the social sciences, ensured that we had a diverse range of perspectives and input into the study design. The first and second (DSS) authors remained conscious of how our positionalities would influence the lens through which we interpreted and analyzed the data. We are both adult British women without children and from outside of East London, and so have different lived experiences from the participants. However, we both have experience of working with children in our academic and professional careers. As adult researchers, we could not dissolve the age-based power differences between ourselves and the participants, but rather attempted to use this position to advocate for children’s voices (Caldairou-Bessette et al., 2020). During analysis, we discussed how our ages and cultural and educational backgrounds may have impacted how we made sense of the data, particularly for drawings which can be more challenging to interpret. Our use of manifest content analysis was an attempt to give participants ‘interpretive authority’, staying close to their words and depictions to avoid mis- or over-interpretation (Carter and Ford, 2013). That said, we came to view our findings as “sensitive and reflexive co-construction” (Carter and Ford, 2013, p. 10) between ourselves and the participants.

## 3 Results

### 3.1 Characteristics of the sample population

Table 2 displays the demographic characteristics of the participants. 48.47% of participants were boys, 46.43% girls and 2.55% identified as ‘other’. The sample was ethnically diverse, with roughly equal numbers of participants across year groups (Table 2). Below, we first outline the findings of RQ3 and RQ4 as they contextualize the findings of RQ1 and RQ2. To aid interpretation, we have included tables to display the percentage of participants who reported the themes across research questions.

**Table 2:** Participant demographic information.

	Boy *n* (% of total sample)	Girl *n* (% of total sample)	Other *n* (% of total sample)	Prefer not to say *n* (% of total sample)	Total *n* (% of total sample)
Ethnicity					
Arab	0 (0)	1 (0.51)	1 (0.51)	0 (0)	2 (1.02)
Asian	29 (14.80)	28 (14.29)	1 (0.51)	1 (0.51)	59 (30.10)
Black	13 (6.63)	9 (4.59)	0 (0)	0 (0)	22 (11.22)
Mixed Ethnicity	8 (4.08)	9 (4.59)	1 (0.51)	0 (0)	18 (9.18)
White	34 (17.35)	30 (15.31)	2 (1.02)	3 (1.53)	69 (35.20)
Other	0 (0)	2 (1.02)	0 (0)	0 (0)	2 (1.02)
Unknown	11 (5.61)	12 (6.12)	1 (0.51)	0 (0)	24 (12.24)
Year group					
Year 3	37 (18.88)	33 (16.84)	3 (1.53)	2 (1.02)	76 (38.78)
Year 4	29 (14.80)	31 (15.82)	2 (1.02)	0 (0)	61 (31.12)
Year 5	29 (14.80)	27 (13.78)	0 (0)	3 (1.53)	59 (30.10)

### 3.2 Content of worries (RQ3)

**Figure 2:**
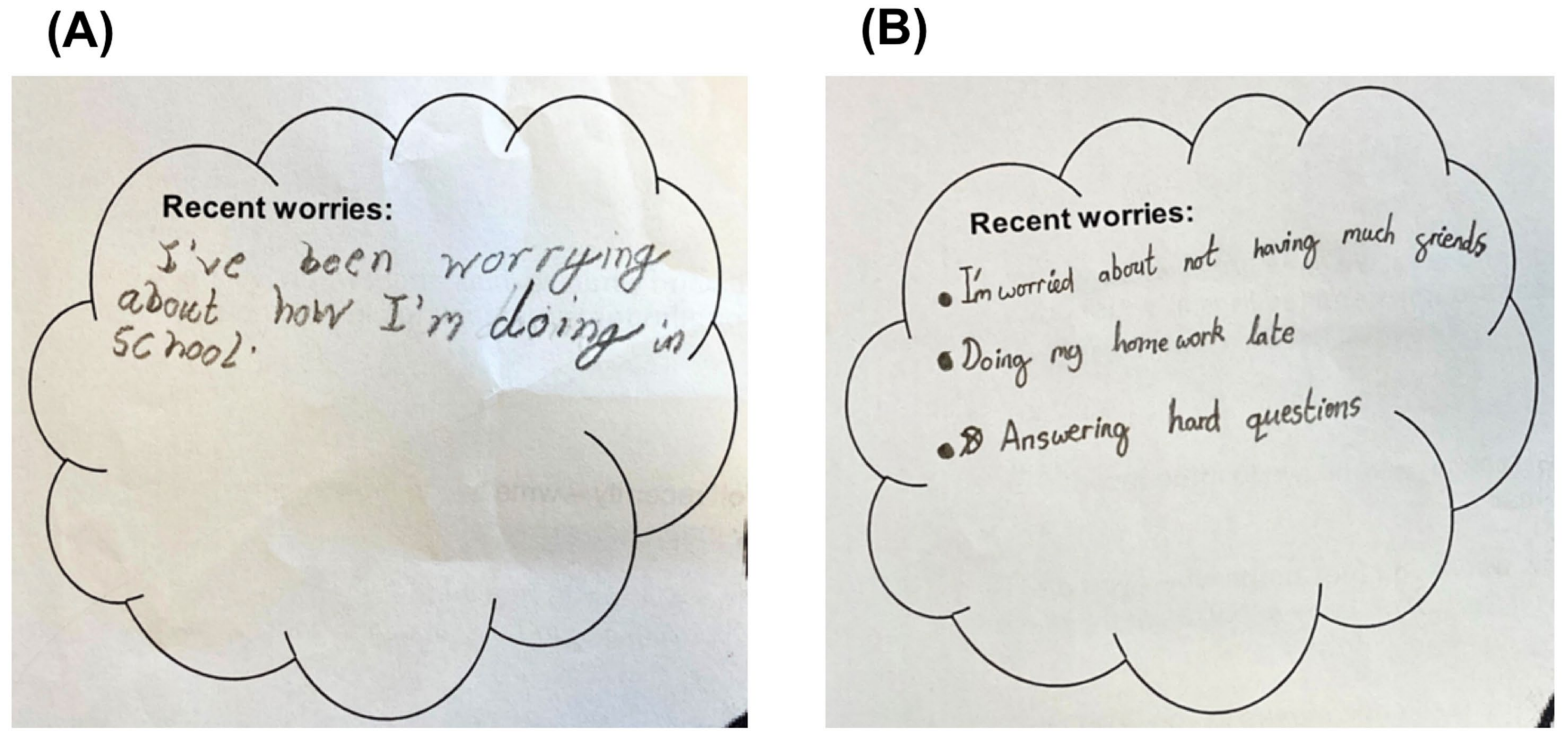
**(A,B)** Examples of children’s worry responses.

Table 3 shows the 11 worry themes derived from participants’ written responses (Figure 2). Detailed codes within each worry theme are included in Supplementary Table S1. The most common worry related to Education, largely expressed as anxiety about academic performance. The next most common worries were Social relationships and interactions, followed by Physical health (of the self and others) and Family and the home.

**Table 3:** The worry themes derived from participants’ responses.

Worry themes	Subthemes	Description of themes	Total *n* (*% of participants)
Education	Academics	Anxiety around completing academic work and performing well, as well as disappointing others through underperformance.	75 (38.27)
	The school environment	Concerns about going to school, getting things wrong at school, relationships with teachers, handwriting and pen licenses.	
Social relationships and interactions	Friendships and peer relationships	Concerns about losing friends, not having many friends, falling out with friends, being bullied or violence and conflict with peers.	68 (34.69)
	Negative interactions and isolation	Concerns about feeling left out or isolated, receiving discipline from others or having conflict with others.	
Physical health	The self	Concerns about diet, being ill, getting hurt or injured, experiencing pain or starting menstruation.	39 (19.90)
	Others	Concerns about the physical health of family members or family getting hurt.	
Family and the home	Family	Concerns about missing or losing family members, parents separating, being fearful of parents, feeling unloved by family or having conflict with siblings.	34 (17.35)
	The home environment	Concerns about housing situations and responsibilities at home.	
Public and private self-consciousness	Public	Concerns about performing in front of others or performance or humiliation in sports.	31 (15.82)
	Private	Concerns about personal failure, self-consciousness, self-image or making mistakes.	
	Both	Concerns which could be perceived as public or private, including about personal appearance, confidence or being late.	
Fears		Expression of fears such as spiders, the dark, monsters/horror or being kidnapped.	30 (15.31)
Death and grief		Fear about one’s own death, the death of family or pets or grief from the death of family members.	17 (8.67)
Change and uncertainty about the future		Concerns about growing up and one’s future, secondary school or university, or anxiousness about change and running out of time.	14 (7.14)
Mental health and emotional wellbeing	The self	Expression of sadness or other negative emotions.	5 (2.55)
	Others	Concerns about wellbeing of family members.	
Global and societal concerns		Concerns about the people of Turkey and Syria following the earthquake or global warming.	2 (1.02)
No worries		Responses indicating that the participant had no worries.	12 (6.12)
Unclear or ambiguous		Concerns where the content of the worry was ambiguous, could not be read or did not fit into a specific category.	22 (11.22)
Invalid		Missing responses.	2 (1.02)

*As participants were not limited in the number of worries they reported, the percentages do not add up to 100%.

### 3.3 Somatic experiences of stress (RQ4)

**Figure 3:**
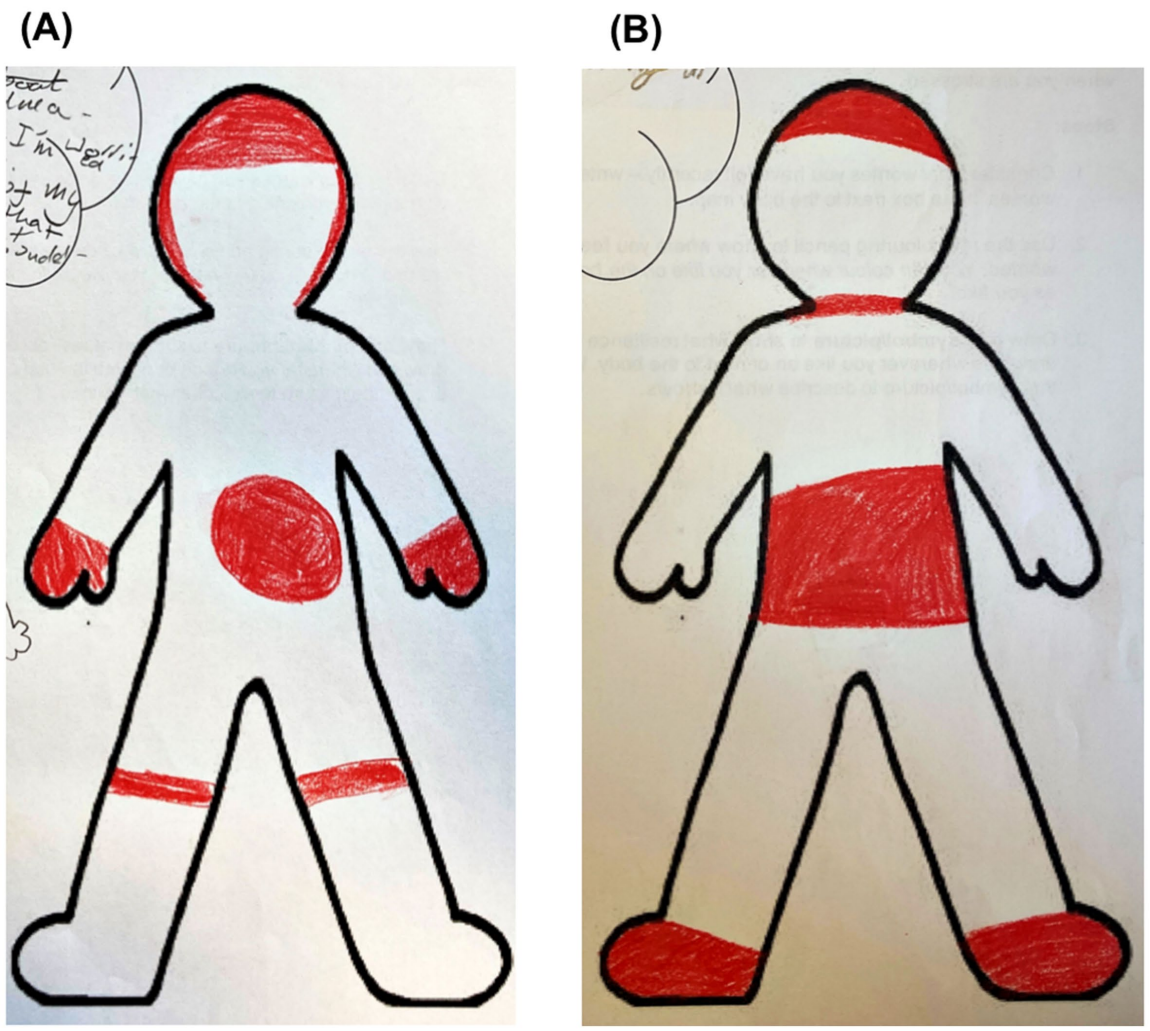
**(A,B)** Examples of children’s coloring of their somatic experiences of stress.

This section displays the results from 162 participants who completed the second version of body mapping (Figure 3). Participants displayed a range of somatic experiences associated with recent stress, resulting in 51 body part categories represented across the body maps. Although largely connected to specific body parts, 5.55% of participants depicted recent stress as a whole-body experience. Table 4 displays an overview of the body parts, which account for those colored by at least 5% of participants, categorized into areas of the body.

**Table 4:** Body parts associated with recent stress.

Body part	Specific areas	Total *n* (*% of participants)
Head	Top of head, whole head, brain region, forehead, top half of head	120 (74.07)
Hands	Both hands, right hand, left hand, fingers, thumbs, left thumb	71 (43.83)
Abdomen/Stomach	Abdomen, stomach, middle of stomach, lower pelvis	66 (40.74)
Feet	Both feet, left foot, right foot, toes	61 (37.65)
Legs	Both legs, both knees, left leg, right leg, right knee	46 (28.40)
Chest	Middle of chest, left side of chest, right side of chest	42 (25.93)
Arms	Both arms, right arm, left arm	31 (19.14)
Throat		21 (12.96)
Torso	Entire torso, left side, right side	13 (8.02)

Body parts accounting for <5%: mouth, eyes, nose, cheeks, sides of the face, shoulders, wrists, groin area, ankles, entire body.

*As participants were not limited in the number of body parts they could color, the percentages do not add up to 100%.

The head was the most frequently colored body part (74.07%). Whilst the majority of this was the top of the head (49.38%), the entire head was colored by 22.22% of participants. Of those who colored the top of the head, 4.94% colored the ‘brain region’ by representing cloud-like shapes in the top of the head or labeling their coloring as ‘brain’. One participant clarified next to their body map that “I get stressed in the brain and head.” The hands were the second most frequently colored region (43.83%), with 30.23% of participants coloring both hands and fewer coloring only the right (6.17%) or left (3.09%) hands, the fingers (2.47%) or the thumbs (1.85%). One participant drew a test paper in their left hand. The abdominal/stomach area (40.74%) was the third most frequently colored body part, with 6.17% of participants representing the middle of the stomach, often with a small circle. One participant drew a butterfly in the abdominal area of their body map, reminiscent of ‘butterflies in your stomach’.

The fourth most frequently colored area was the feet (37.65%), with 27.78% of participants coloring both feet and fewer coloring only the left (4.32%) or right (3.09%) foot or just the toes (2.47%). The legs were colored by 28.40% of participants; 13.58% of participants colored both legs, whilst only 4.94 and 4.32% colored only the left or right, respectively. Either both knees or just the right knee (categorized under legs) were colored by 5.56% of participants. The chest area was colored by 25.93% of participants. 7.41% of participants drew hearts; the majority of these were positioned in the chest, but one participant drew this in the throat. One participant, who drew a heart in the left side of the chest, wrote “here.”

### 3.4 Resilience (RQ1)

Table 5 displays the resilience themes derived from participants’ drawings and descriptions. Detailed codes within each resilience theme are included in Supplementary Table S2. These themes fall into two categories related to how resilience is conceptualized (concepts of resilience) and the factors which contribute to it (socioecological resilience factors). Descriptions of each of these categories and included themes are expanded upon below.

**Table 5:** The resilience themes derived from participants’ drawings.

Resilience themes	Subthemes	Description of themes	Total *n* (*% of participants)
Concepts of resilience			
Conceptual definitions		Concepts of resilience mainly related to personal abilities, including perseverance, bouncing back and personal strength.	50 (25.51)
Symbolic reminders of resilience: nature and fantasy		Elements of nature and imagination to symbolize resilience, including animals, mythical creatures, flowers and rainbows.	17 (8.67)
Metaphorical depictions of personal strength		Abstract depictions of resilience as a personal quality, represented by superheroes, Bear Grylls, space imagery, for example.	14 (7.14)
Socioecological resilience factors			
Individual level	Interests and leisure	Hobbies and leisure activities, such as sports, videogames and media and reading.	57 (29.08)
	Personal skills and qualities	Personal resilience-enhancing skills and qualities, including maintaining a positive mindset, reflecting on emotions and self-soothing.	
Interpersonal level	Social networks	Important social connections, including family and friends	34 (17.35)
	Supportive interactions	Interpersonal interactions, particularly providing and receiving support from others, including from pets.	
Organizational level		Education as a factor promoting resilience.	2 (1.02)
Community level		Factors at the community level which support resilience, including faith and access to basic needs.	6 (3.06)
Negative descriptions		Responses which were negative, such as negative emotions and experiences.	9 (4.59)
Unclear or ambiguous		Concerns where the content of the drawing was ambiguous, could not be interpreted or did not fit into a specific category.	14 (7.14)
Invalid		Missing responses.	10 (5.10)

#### 3.4.1 Concepts of resilience

##### 3.4.1.1 Conceptual definitions

**Figure 4:**
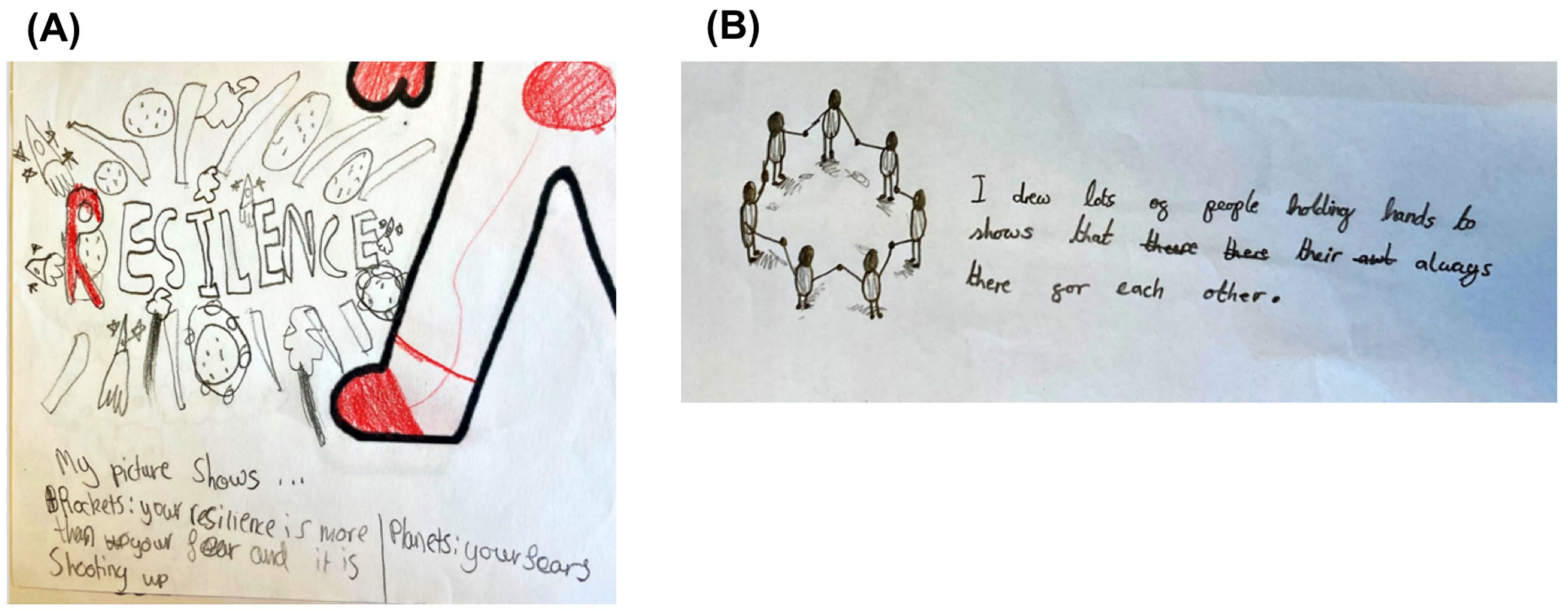
**(A,B)** Examples of children’s drawings of concepts of resilience.

Concepts of resilience largely related to personal qualities which participants associated with resilience. Perseverance was the most common understanding of resilience, with participants often referring to the idea of ‘not giving up’. This was sometimes represented through nature-based images such as “a fish who has to push through a current” or more human-made images like a “shield represent[ing] not breaking (giving up).” This was also sometimes depicted through personal experiences, particularly work; one participant depicted a figure at a desk and wrote “me doing my work and not giving up.” Similarly, participants described Bouncing back, again in personal contexts, e.g., of a sports injury or following “a friend being rude to you.” Some participants described resilience as Overcoming sadness, with one participant drawing multiple happy and sad faces inside each other labeled “you are happy you turn sad then you turn happy again.” Positive emotions also emerged; several participants drew smiling faces, sometimes accompanied with positive emotions written, such as “happy emotions” and “joy.” Two participants depicted getting back up again, including one participant describing “if you fall while riding your bicycle try riding it again.” One participant described resilience as an active personal choice: “when I feel sad I imagine that it is on my shoulders then I boing it off.” Others emphasized Collective support. One participant drew a group of stick figures holding hands, explaining that “I drew lots of people holding hands to shows that their always there for each other” (Figure 4). The interactional nature of resilience was also shown through depictions of Love, such as drawings of love hearts. Praise from others, for example for “good work,” was also depicted.

##### 3.4.1.2 Symbolic reminders of resilience (nature and fantasy)

Several participants portrayed nature and imaginative concepts to represent resilience. Animals were depicted most frequently, particularly dogs and cats. Three participants drew Mythical creatures, all of which were dragons. Elements of nature included Flowers and Rainbows. More broadly, one participant illustrated Planet Earth, with the caption “the earth is the planet I live on so it makes me happy because my world is my life.” These symbols of nature and fantasy served as meaningful reminders and representations of resilience for participants.

##### 3.4.1.3 Metaphorical depictions of personal strength

Metaphorical depictions of resilience included Superheroes, such as drawings of Wonder Woman. Attributes of superheroes were “being brave” and “sav[ing] people.” Similarly, one participant depicted Bear Grylls. Imagery of space also featured as a metaphor for overcoming difficulties, including a scene of rockets (representing resilience) and planets (representing fear) to show “your resilience is more than your fear and it is shooting up” (Figure 4). Flying in the sky, Opening a door and Climbing stairs were also depicted.

The following section outlines the themes of resilience factors which emerged, situated within the socioecological framework.

#### 3.4.2 Socioecological resilience factors

##### 3.4.2.1 Individual level

At the individual level, the subthemes of Personal skills and qualities and Interests and leisure emerged.

Personal skills and qualities: Some participants conceptualized resilience as an internal ability to overcome adversity. The most common quality portrayed was Maintaining a positive mindset, such as through “see[ing] the bright side of things” and “think[ing] happy thoughts.” Similarly, one participant reflected on their Self-soothing practice of “when I try to calm myself down.” Reflecting on one’s emotions was also depicted by one participant, who drew a smiling figure with a speech bubble saying “how am I feeling?”

**Figure 5:**
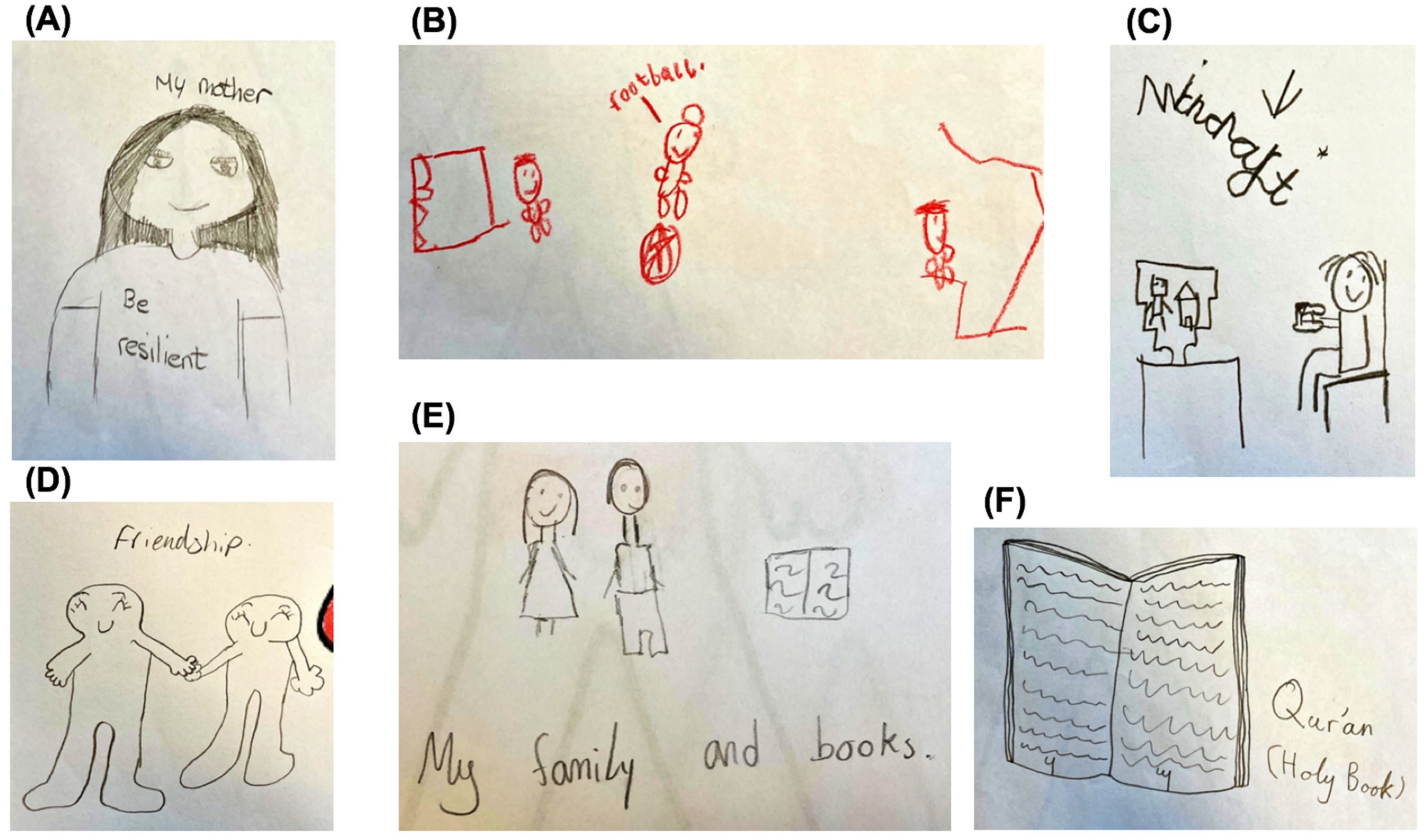
**(A–F)** Examples of children’s drawings of resilience factors.

Interests and leisure: Participants represented various personal hobbies and leisure activities, most commonly Sports, and football in particular (Figure 5). Videogames and media were also frequently depicted, including references to Minecraft, Fortnite, Rocket League and PS4 (Figure 5). One participant described that “movie, games helps me calm down.” Engagement in the arts, such as drawing and painting, was also cited as a stress relief, one which “makes me calm down and makes me feel happy” and helps “let out all of my worries and feelings.” Reading, Toys and Shopping were also mentioned as supportive leisure activities and possessions.

##### 3.4.2.2 Interpersonal level

The interpersonal level includes two subthemes of Social networks and Supportive interactions.

###### 3.4.2.2.1 Social networks

Several participants drew figures of their Family and friends, including specific depictions of Mothers (Figure 5). Beyond visualizing these relationships, some participants described Support from parents and other family members and Playing with parents. Regarding friendship, participants described Support from friends and Socializing and making up with friends. For example, one participant described that “when I am stressed I like to talk to my friends.” A Home was also depicted by two participants. Given many interactions and experiences occur within the home environment, this was interpreted as reflecting social network.

###### 3.4.2.2.2 Supportive interactions

Participants conveyed knowledge of the interpersonal and interactional dynamics of resilience. Supporting others emerged most commonly within this subtheme; for example, one participant depicted a figure asking another “are you OK?” with the caption “when I help a person when they are sad.” Seeking and receiving support from others was also conveyed in the context of feeling sad, with one participant noting “when I feel sad I talk to a person about something.” Similarly, two participants drew images of their Pets, which children often turn to for comfort and emotional support. One participant wrote that “when I feel worried I like to play with my dog to calm me down.”

##### 3.4.2.3 Organizational level

Only two examples of resilience factors at the organizational level emerged, both of which related to Education. In one case, engagement in a school subject was described as a stress relief, with a figure at a desk and an “English” book drawn and the caption “when I’m stressed I do English.” Another participant drew a “school,” suggesting school as a resilience-supporting environment, but provided little detail regarding the nature of this.

##### 3.4.2.4 Community level

Few examples of resilience factors at the community level emerged. Spiritual resources were the most common factor; three participants depicted symbols of Islam, including the Qur’an and ‘Allah’ in Arabic (Figure 5). One participant also portrayed Physical resources such as “clothes,” “water and “food.”

### 3.5 Differences by gender (RQ2)

At the Individual level, gender differences were found within the theme of Interests and leisure. Boys depicted Sports (14.89% vs. 3.30%, 14 vs. 3 responses) and Videogames and media (8.51% vs. 1.10%, 8 vs. 1 responses) more often than girls. However, within this theme, only girls drew Toys (3.30%, 3 responses) and Engagement in the arts (8.79%, 8 responses). The theme of Interpersonal level: Social networks was more commonly expressed by girls than boys (19.78% vs. 6.38%, 18 vs. 6 responses). This included aspects of friendship, such as socialising and playing with friends, and family support.

## 4 Discussion

### 4.1 Summary of key findings

This study employed a novel body mapping tool to explore resilience, recent worries and somatic stress among children aged 7–10 in East London. Aligned with existing literature, worries around education and social relationships were prominent. Somatic experiences of stress reflected common complaints in children, yet body mapping also revealed unique experiences. Resilience was shown to be an interplay of factors across socioecological levels, with personal resources and strong social relationships particularly valued by participants. We also found gender differences regarding resilience factors at the individual and interpersonal levels. Our qualitative research design enabled us to gather data on perceptions and experiences which can be challenging to capture with quantitative methods, particularly with children.

### 4.2 Situating findings within existing literature

Findings of RQ1 revealed that children commonly defined resilience as an individual capacity to persevere, which aligns with earlier dominant notions of resilience which remain widely held. However, children also used abstract imagery to define resilience, such as through depictions of nature and animals. Whilst some of these depictions were only created by a small number of participants, such as flowers, rainbows and Planet Earth, examining these exceptions can provide insight into diverse and nuanced perspectives (McPherson and Thorne, 2006). For instance, participants associating resilience with nature is not commonly reported in the literature, but alludes to it as an entwinement of mind, body and environment. We can then draw parallels with the growing evidence of the benefits of nature-based interventions, including arts-in-nature, for promoting mental health and resilience in CYP (Moula et al., 2022; Obeng et al., 2023; Overbey et al., 2023). Children often make sense of the world in abstract and imaginative ways, and so drawing enabled children’s shared and unique “ideas, beliefs, and metaphors” (Pridmore and Bendelow, 1995, p. 473) to become part of the research process. Through acknowledging these less common findings, we can gain a deeper understanding of diverse concepts of resilience that are important to children.

**Figure 6:**
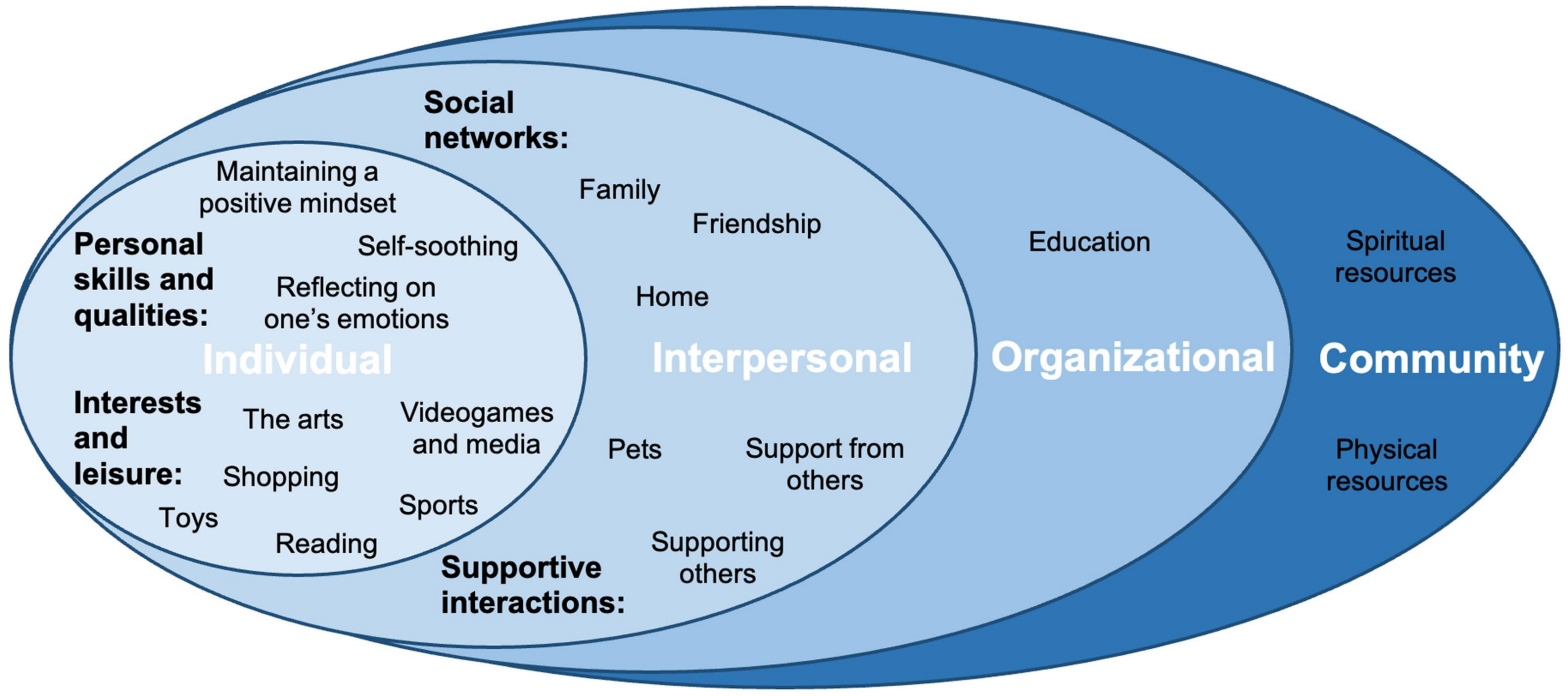
A diagram to show participants’ responses on resilience factors embedded within a socioecological framework. These emerged from the individual up to the community level.

In the analysis of resilience factors (RQ1), we situated participants’ responses within a socioecological framework (RQ1), whereby resilience develops from “a hierarchically organized set of protective systems that cumulatively buffer the effects of adversity” (Roisman et al., 2002, p. 1216). Under15standing the factors operating at different levels of influence can inform strategies that promote resilience (Twum-Antwi et al., 2020). The majority of participants’ responses emerged at the individual and interpersonal levels (Figure 6). Sports and other hobbies were frequently cited, which play a role in the development of instrumental and social skills and social networks (Gilligan, 2000). Although hobbies reflect individual interests, their benefits can relate to their social and relational qualities, for example participation in sports has been shown to increase social competence and empathy in adolescents (Caldarella et al., 2019) and arts activities has been found to improve relationship building and a sense of belonging in CYP (Zarobe and Bungay, 2017).

At the interpersonal level, family, friends and pets were commonly depicted. These factors underscore the significance of social support in fostering resilience (Gartland et al., 2019). Research suggests that positive family and social support systems are associated with resilient outcomes in children experiencing social adversity (Gartland et al., 2019). Additionally, pets have been shown to increase children’s resilience and self-worth (Purewal et al., 2017), including the reduction and prevention of depressive symptoms (Gadomski et al., 2015). These findings are corroborated by a recent meta-analysis which found that social support-based interventions yielded the largest positive effects for action-based resilience outcomes (i.e., functional and/or performance-related activities in relation to the intervention) (Liu et al., 2020). This highlights the value of interventions which facilitate supportive relationships between children and important people in their lives, including family, peers and educators (McDonald et al., 2019).

At the organizational level, whilst only two participants described education in relation to resilience, highlighting this as an exception reveals the stark contrast with how commonly school was instead described as a worry. This suggests educational settings as ideal sites for interventions to improve psychosocial wellbeing. For example, school-based programmes focused on mindfulness have been shown to help students overcome social, emotional and behavioral problems (Schonert-Reichl and Lawlor, 2010). The limited findings on community and policy levels are unsurprising given the young age of participants. However, the inclusion of faith as a community factor is concurrent with research suggesting religion to be an important coping strategy and protective factor for many communities (Foy et al., 2011; Pielock et al., 2016; Mhaka-Mutepfa and Maundeni, 2019). Our findings mirror one systematic review which found less evidence for school and community factors associated with resilient outcomes in children experiencing social adversity compared with individual factors (Gartland et al., 2019). Whilst our intentional focus on children in East London contributes to the growing body of literature on resilience in children from diverse backgrounds, there is scope for further research which investigates these wider socioecological factors.

Gender differences in the prevalence and trajectories of mental health difficulties and types of resilience resources that CYP use have been explored extensively (Rescorla et al., 2007; Martin and Hadwin, 2022; Yoon et al., 2023; Mar et al., 2024). Our results found gender differences in resilience factors that align with research on the gendered socialization of boys and girls (RQ2). For example, relational resilience, whereby resilience arises through fostering mutually empathic connections (Jordan, 2023), is believed to be more common in girls who are socialized to see themselves as relational entities (Miller, 1986). Girls in our study more often depicted interpersonal coping strategies (e.g., family and peer relationships). Boys chose individualistic, competitive and task-oriented strategies (e.g., sports and videogames), which aligns with research on their socialization (Frydenberg and Lewis, 1993). However, access to recreational resources is an important community protective factor for resilience (Zolkoski and Bullock, 2012) and the peer acceptance gained through participation in sports may contribute to feelings of self-esteem in adolescents (Daniels and Leaper, 2006). These peer and community aspects of sports may explain the prevalence of team sports described by boys in our sample. These results emphasize the importance of children having access to a range of resources that go beyond gender stereotypes, as this may constrain children’s capacity to develop resilience. Gender-informed and gender-specific mental health interventions may be more effective at supporting CYP’s mental health and resilience (Friedrich et al., 2010; Herrmann et al., 2024). However, few trials of resilience-supporting interventions assess their gender-specific effects, leaving uncertainty about whether targeting gender-related protective factors through interventions could lead to better mental health outcomes (Dray et al., 2017). Future research could elucidate this by incorporating analysis of the effect of interventions by gender. There is also a need for more research which explores risk and protective factors in the lives of transgender, non-binary, and gender diverse CYP to inform tailored interventions (Johns et al., 2018; Chew et al., 2020), particularly given existing research which highlights that these CYP face increased risk of emotional distress, suicidal ideation and severe mental health conditions (Eisenberg et al., 2017; Holt et al., 2023).

The results of RQ3 revealed 11 main worry themes identified from children’s responses. Many of these align with existing literature on the worries of children aged 12 and below, including school, health, social relationships and death (Silverman et al., 1995; Muris et al., 1998, 2000; Christie and MacMullin, 2016). School and academic performance emerged as primary worries, consistent with studies highlighting these as among the most common areas of worry in primary school-aged children (Silverman et al., 1995; Muris et al., 1998, 2000) and young people during the COVID-19 pandemic (Cauberghe et al., 2021; Shukla et al., 2023). Social relationships and interactions was the second most common worry category, reflecting the substantial role that managing social relationships plays in early life stress (Cheetham-Blake et al., 2019). This relational aspect of worry emerged across categories, such as concern about the health of others and public and private self-consciousness. This co-exists with the finding of social networks and supportive interactions as resilience factors, with research suggesting that learning to navigate social relationships and maintaining strong peer and adult-child relationships can help build resilience (Waaktaar et al., 2004; Graber et al., 2016; Twum-Antwi et al., 2020).

The body maps captured 51 body parts reflecting somatic stress symptoms (RQ4). The top of the head was most frequently colored, followed by the hands and abdomen. Headaches and abdominal pain are two of the most common somatic complaints among children and adolescents (Goodman and McGrath, 1991; King et al., 2011), and both have been associated with pressure to perform academically (Passchier and Orlebeke, 1985; Randall et al., 2019). This aligns with the high frequency of worry responses in our study about academic performance. The prevalence of the abdomen and chest also reflect somatic symptoms and measures used to detect anxiety in common diagnostic and scale tools. Whilst the association of stress with the head and hands is less common in these tools, these body parts are associated with common somatic traits of stress and anxiety (e.g., headaches, sweating, shaking and clenching) and were also commonly represented as such in a body mapping study on embodied experiences of anxiety (Vaughan et al., 2023). The prevalence of the hands could also reflect academic anxiety and physical discomfort from writing, for example one participant drew a test paper in their hand. 12 body parts were unique to individual body maps and 45 were found on ten maps or less, supporting the idea that embodied experiences of stress can be highly individualized (Vaughan et al., 2023).

### 4.3 Situating findings within the lived experience of children

In the absence of verbal data, AM led an activity and discussion with an advisory group of six students in Years 3, 4 and 5, from a primary school in East London, to discuss the findings on resilience. After discussing how they would define resilience, the students were shown the resilience topics and asked to select six which resonated with them and visually represent these on a ‘coat of arms’. Regarding concepts of resilience, the idea of perseverance emerged, with one child describing resilience as “when life is hard, just keep going.” Whilst only one participant in the study depicted Planet Earth as a symbolic reminder of resilience, it resonated with two children from the advisory group, one of whom related it to her love of nature and the outside world.

For resilience factors, videogames were a favourite hobby for several children, one of whom particularly enjoyed playing with their friends. Whilst the impact of videogames on children’s health is often perceived as negative, there is growing evidence of their positive psychological effects (Johnson et al., 2013), including lower prevalence of mental health difficulties and peer relationship problems (Kovess-Masfety et al., 2016). Team sports like football were also commonly described by the group, and for one child their enjoyment was related to being good and football and the sense of achievement that came with this. At the interpersonal level, several children described family, friends and pets as sources of resilience. The home was also depicted, with one child expressing that “my family is my home.” Compared with study participants, all children in the advisory group expressed that they enjoyed school at times, when prompted during the discussion.

### 4.4 Implications for future research and practice

Our method draws upon the draw and write approach (Gauntlett and Horsley, 2004), which helps children convey complex ideas and recall events (Horstman et al., 2008). Compared with traditional research methods, drawing may be less anxiety-inducing for children (de Jäger et al., 2016), even for sensitive topics (Hill et al., 1996; Horstman et al., 2008). However, criticisms of draw and write include the challenges of interpretation and analysis of children’s drawings and its limited sensitivity to social context (Backett-Milburn and McKie, 1999; Angell et al., 2014). Whilst AM and DSS referred back to participants’ responses during analysis, some clarity of meaning may have been lost due to reliance on researcher interpretations. Whilst we carried out consultation with an advisory group, a refinement of our method could incorporate the ‘draw-write-tell’ approach (Angell and Angell, 2013), which combines drawing with verbal interpretations. It has been argued to more effectively equalize adult-child power imbalances and centralize children’s voices (Angell et al., 2014). Further research incorporating this approach could be done to evaluate the efficacy of the body mapping method utilized in this study for understanding children’s subjective experiences.

Our study fits within a growing body of literature using body mapping to explore embodied symptoms and experiences, including embodied anxiety and non-anxiety in a general population sample (Vaughan et al., 2023), embodied feelings following a surfing intervention for children with autism (Britton et al., 2020) and as an assessment of embodied trauma in refugees and asylum-seekers (O’Brien and Charura, 2023). With its origins in art therapy (Solomon, 2002), body mapping has been used as treatment for trauma and dissociation in children (Santen, 2007, 2015). Combined, this points to the potential for future research to explore the clinical application of this body mapping approach. For example, authors have suggested that body mapping could be applied to the diagnosis and treatment of anxiety (Vaughan et al., 2023) and assessment of embodied trauma (O’Brien and Charura, 2023). Digital and web-based approaches could be combined with body mapping to offer rapid and dynamic assessment of children’s embodied experiences (Edwards, 2021; Ludlow, 2021; Ticho, 2021; Shanneik and Sobieczky, 2023). This reduced reliance on drawing and movement may make the method more inclusive for individuals who are not comfortable with drawing and those with reduced mobility. Body mapping is also a promising educational tool (Chenhall et al., 2013; Senior et al., 2014; Helmer et al., 2015; Lys et al., 2018; Cook et al., 2022; Henderson et al., 2023). As our version is simple and suitable for large groups, it holds potential as a tool for teachers and others working with children to explore embodied experiences across a range of issues, including mental health (Barnes et al., 2024). Further research is needed to evaluate these potential applications of body mapping.

## 5 Limitations

This study has several limitations. First, whilst we had a large and diverse sample, the small age range of participants prevented age-based comparisons. This small age range also means that our findings cannot necessarily be generalized to older or younger children, yet the consistency of our findings with existing research suggests some transferability to other age groups (Tracy, 2010). There is scope for further research using body mapping with more diverse age groups. Second, the lack of complementary verbal data may have led to misinterpretation of participants’ intended meanings. Whilst challenging due to our large sample size, post-body mapping interviews would help deepen analysis and ensure accurate representation of meaning (Vaughan et al., 2023). Third, although drawing has been described as a “cultural practice” in childhood (Christensen and James, 2008, p. 162), not all children enjoy drawing or find it easy. Although anecdotally, participants often enjoyed body mapping, some children may have felt self-conscious or excluded. Fourth, we used a premade body map outline, which differs from traditional body mapping and may have constrained creative expression (Britton et al., 2020). However, given the young age of participants, this aided understanding of the activity as well as consistent analysis. Fifth, despite efforts to encourage independent work, participants may have been influenced by their peers when completing the activity.

## 6 Conclusion

Understanding the factors which children view as important for their resilience can inform interventions that support children in navigating and overcoming adversity. Despite limitations such as the lack of verbal data, our body mapping tool offered a simple and accessible way to understand how children perceive worries, experience somatic stress and conceptualize resilience. Although exploratory, our findings suggest that access to personal resources and strong social relationships are valued by children, and the relational aspects of these factors may be particularly important for promoting resilience for this age group. Body mapping holds promise for engaging with children in clinical and educational settings.

## Data Availability

The raw data supporting the conclusions of this article will be made available by the authors, without undue reservation.
