# Supplementary material for: Children’s emerging concepts of resilience: insights from using body mapping in an East London cohort sample of 7-10-year-old children
Source: Front Psychol. 2025 Jan 6;15:1408771. doi: 10.3389/fpsyg.2024.1408771 (PMC11743963; doi:10.3389/fpsyg.2024.1408771)
Supplement: Supplementary file 1 [file Table_1.DOCX]

**Supplementary Table 1.** Subthemes and codes classified under each of the worry themes apart from ‘no worries’ and ‘invalid’.

| **Worry themes** | **Subthemes (if applicable)** | **Codes** |
| --- | --- | --- |
| Education | Academics | Academic anxiety, academic achievement, disappointing others through academic underperformance (teachers/family), religious exams, learning |
|  | The school environment | School, getting things wrong at school, change, concern about handwriting, not receiving a pen license, relationships with teachers |
| Social relationships and interactions | Friendships and peer relationships | Anxiety about friendships, being bullied, friendship, violence with peers, friendship troubles, concern about friends, conflict with peers, peer pressure |
|  | Negative interactions and isolation | Being alone, being lost, discipline, feeling left out, abandonment, loneliness, social disagreements, being shouted at, feeling left out in sports, others being rude, sanctions, sanctions in sports, scared to ask to go to the toilet |
| Physical health | The self | Personal health, having an illness, parts of the body, getting hurt, injuries, menstruation |
|  | Others | Family health, family getting hurt |
| Family and the home | Family | Family, missing family members, family dynamics, concern about siblings, losing material items because of sibling, separation from family members, fear of parents, feeling unloved by family, left at home with baby sibling, playing football with sibling |
|  | The home environment | Home, responsibilities, car breakdown |
| Public and private self-consciousness | Private | Doing something wrong, failure, making mistakes, negative self-image, not knowing something, self-consciousness |
|  | Public | Performing, performance in sports, humiliation in sports, people talking about their hair |
|  | Both | Personal appearance, being late, confidence |
| Fears |  | Spiders, the dark, horror, being kidnapped, flying, trouble with sleep, burglary, fear of car travel, the dentist, heights, rollercoasters, natural disasters, plane crash, swallowing a tooth |
| Death and grief |  | Mortality, death of family member, delayed grief, fear of family member dying, fear of pet dying |
| Change and uncertainty about the future |  | Growing up, secondary school, anxiety about the future, running out of time, someone getting hurt, trying something new, things happening suddenly, future academic studies |
| Mental health and emotional wellbeing | The self | Sadness, negative emotions |
|  | Others | Family wellbeing |
| Global and societal concerns |  | Concern about the people of Türkiye and Syria following earthquake, global warming |
| Unclear or ambiguous |  | Sports, football team losing a match, buying things, hallucinations, hospital |
