# Supplementary material for: Children’s emerging concepts of resilience: insights from using body mapping in an East London cohort sample of 7-10-year-old children
Source: Front Psychol. 2025 Jan 6;15:1408771. doi: 10.3389/fpsyg.2024.1408771 (PMC11743963; doi:10.3389/fpsyg.2024.1408771)
Supplement: Supplementary file 2 [file Table_2.DOCX]

**Supplementary Table 2.** Subthemes and codes classified under each of the resilience themes apart from ‘unclear’, ‘invalid’ and ‘negative descriptions’.

|  | **Resilience themes** | **Subthemes (if applicable)** | **Codes** |
| --- | --- | --- | --- |
| Concepts of resilience | Conceptual definitions |  | Perseverance, positive emotions, bouncing back, love, not giving up with work, overcoming sadness, kindness, bouncing back in sports, collective support, gratitude, personal strength, praise from others |
|  | Symbolic reminders of resilience: nature and fantasy |  | Animals, mythical creatures, rainbows, flowers, Planet Earth |
|  | Metaphorical depictions of personal strength |  | Superheroes, space imagery for overcoming difficulties, Bear Grylls, climbing stairs, flying, opening a door, getting back up again |
| Socioecological resilience factors | Individual level | Interests and leisure | Sports, videogames and media, engagement in the arts, reading, toys, shopping, alone time |
|  |  | Personal skills and qualities | Having a positive mindset, reflecting on emotions, self-soothing |
|  | Interpersonal level | Social networks | Family, friends, mothers, support from parents and other family members, home, pets, socialising with friends, making up with friends, playing with parents, support from friends |
|  |  | Supportive interactions | Supporting others, support from others, seeking and receiving support from others |
|  | Organisational level |  | Education |
|  | Community level |  | Faith, basic needs |
