# Supplementary material for: Children’s emerging concepts of resilience: insights from using body mapping in an East London cohort sample of 7-10-year-old children
Source: Front Psychol. 2025 Jan 6;15:1408771. doi: 10.3389/fpsyg.2024.1408771 (PMC11743963; doi:10.3389/fpsyg.2024.1408771)

Participant ID: _ _ / _ _ / _ _ _ _ **Assessment Year: __**

Exercise: Body mapping

Background:

Stress includes what we feel in our *bodies* and what we *think about* when we are stressed. We want to know more about your thoughts and feelings in your body when you are stressed.

Steps:

1. Consider *what* worries you have felt recently – write two to three recent worries in the box next to the body map.

2. Use the red colouring pencil to show *where* you feel things when you are worried. *You can colour wherever you like on the body and in as many places as you like.*

3. Draw **one symbol/picture** to show what resilience means to you. You can draw this wherever you like on or next to the body. Write **one word** next to this symbol/picture to describe what it shows.

Recent worries:

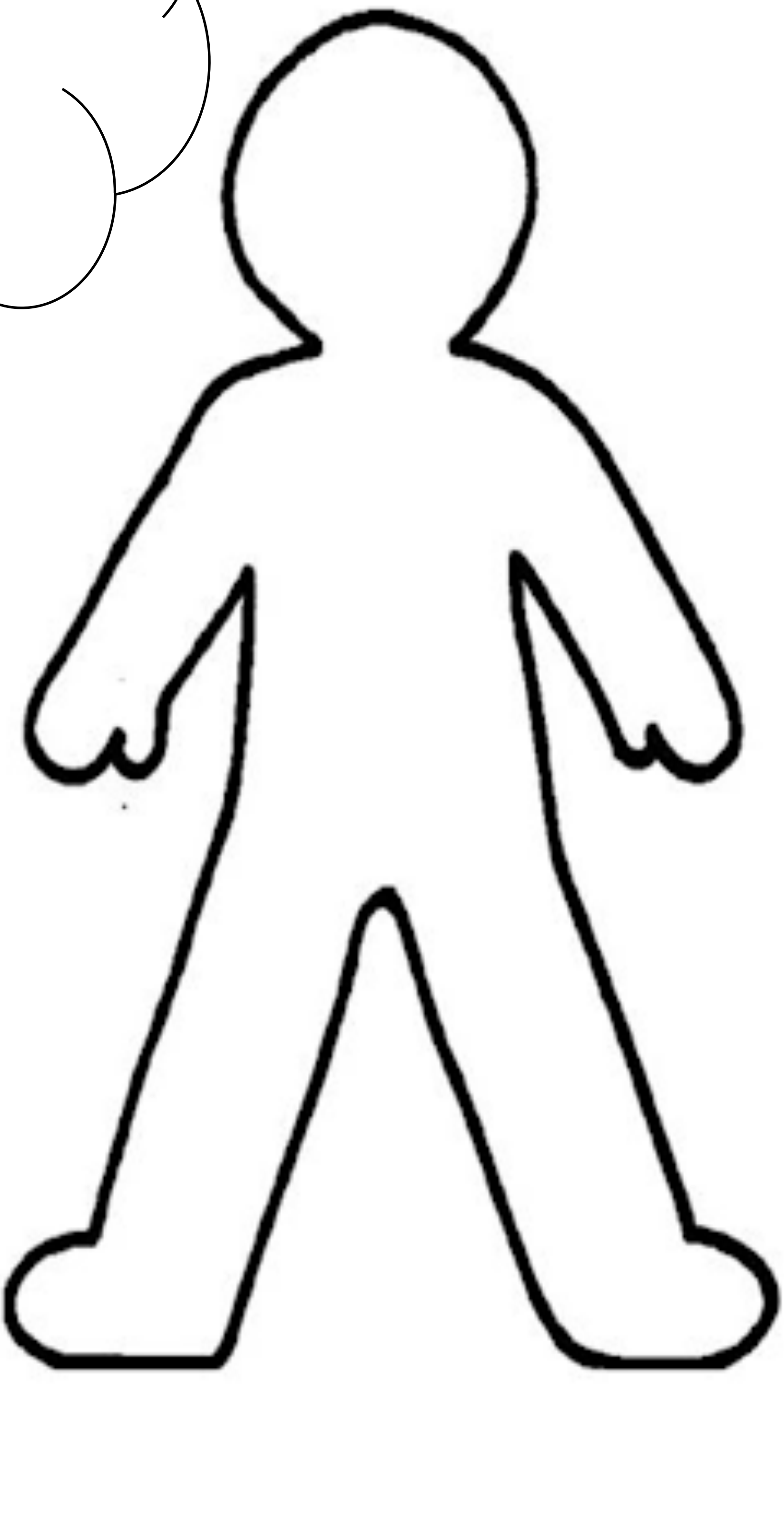

Supplement: Supplementary file 3 [file Data_Sheet_1.PDF]
